# Treatment Patterns and Radical Cystectomy Outcomes in Patients Diagnosed With Urothelial Nonmetastatic Muscle‐Invasive Bladder Cancer in the United States

**DOI:** 10.1002/cam4.70644

**Published:** 2025-02-13

**Authors:** Bernard Bright Davies‐Teye, M. Minhaj Siddiqui, Xiao Zhang, Abree Johnson, Mehmet Burcu, Eberechukwu Onukwugha, Nader Hanna

**Affiliations:** ^1^ Department of Practice, Sciences, and Health Outcomes Research University of Maryland School of Pharmacy Baltimore Maryland USA; ^2^ University of Maryland School of Medicine Baltimore Maryland USA; ^3^ Merck & Co., Inc. Rahway New Jersey USA

**Keywords:** conservative treatment, cystectomy radical, neoadjuvant chemotherapy, treatment outcomes, urinary bladder neoplasm

## Abstract

**Purpose:**

To characterize trends and patterns in treatment characteristics and perioperative outcomes of patients with urothelial muscle‐invasive bladder cancer (MIBC).

**Materials and Methods:**

We utilized the National Cancer Database to assess trends and patterns in treatment modalities (radical cystectomy [RC] with or without neoadjuvant/adjuvant treatments, trimodal bladder‐sparing treatment [trimodal treatment], and others) among MIBC patients diagnosed between 2004 and 2017. We also assessed trends and patterns of short‐term post‐surgery outcomes, including 30‐day and 90‐day mortality, and readmissions.

**Results:**

Among 83,259 MIBC patients, those who received RC, trimodal treatment, and transurethral resection of bladder tumor (TURBT) plus chemotherapy were 34,715 (41.7%), 7,372 (8.9%), and 6,171 (7.4%), respectively. A substantial proportion (29,314; 35.2%) of MIBC patients received other treatments, including TURBT‐only. From 2004 through 2017, the proportion of MIBC patients who utilized guideline‐recommended treatments, whether RC (from 36.4% to 42.8%) or trimodal treatment (from 7.9% to 10.2%), increased. Among those who received RC, there was a substantial increase in neoadjuvant chemotherapy (NAC) utilization, from 7.8% to 29.4%. Conversely, utilization of RC without perioperative treatments decreased from 62.3% to 32.7%. There was a significant decrease in 30‐day (2.8%–1.8%) and 90‐day (7.1%–5.3%) mortality rates among RC recipients.

**Conclusion:**

There was a shift in treatment modalities for MIBC, with increased utilization of RC with NAC. A decrease in post‐surgery mortality rates may indicate improved outcomes, although the unmet need for NAC utilization requires further investigation.

## Precise

This study characterized trends and patterns in treatment characteristics and perioperative outcomes of patients with urothelial muscle‐invasive bladder cancer (MIBC). We utilized the National Cancer Database to assess trends and patterns in treatment modalities (radical cystectomy [RC] with or without neoadjuvant/adjuvant treatments, trimodal bladder‐sparing treatment [trimodal treatment], and others) among MIBC patients diagnosed between 2004 and 2017. There was a shift in treatment modalities for MIBC, with increased utilization of RC with NAC. A decrease in post‐surgery mortality rates may indicate improved outcomes, although the unmet need for NAC utilization requires further investigation.

## 1 Introduction

The National Comprehensive Cancer Network (NCCN), the American Urological Association (AUA), the American Society of Clinical Oncology (ASCO), and the European Association of Urologists guidelines recommend cisplatin‐based neoadjuvant chemotherapy (NAC) plus radical cystectomy (RC) as the standard treatment for individuals diagnosed with clinically staged T2‐T4a, N0, M0 (i.e., T2‐T4aN0M0) muscle‐invasive bladder cancer (MIBC) (hereafter referred to as nonmetastatic MIBC) [1, 2, 3, 4, 5]. The receipt of NAC plus RC is associated with disease downstaging, improved quality of life, reduced risk of disease recurrence, and increased survival in patients diagnosed with nonmetastatic MIBC [1, 2, 4, 6, 7, 8, 9].

More recently, MIBC clinical stage T2‐T4a, N0 or N1, M0 tumor treatment generally consists of the following: (1) NAC plus RC; (2) NAC plus partial cystectomy (PC) for selected patients; (3) RC alone for patients unable to receive chemotherapy; and (4) bladder preservation with concurrent chemoradiotherapy and maximal transurethral resection of bladder tumor (TURBT), also known as TMT [4, 10]. The NCCN guidelines recommend TMT for individuals with MIBC clinical stage T2‐T4a, N0 or N1, M0 tumors who (1) have serious comorbidities or cannot physically complete day‐to‐day activities and are unable to undergo cystectomy (RC or PC), or (2) decline cystectomy. For MIBC clinical stage T2‐T4a, N2 or N3, M0 tumors, the NCCN treatment guidelines recommend: (1) downstaging systemic therapy, or (2) concurrent chemoradiotherapy [4, 10, 11]. These recommendations differ significantly from those for clinical stage T4b, any *N*, M0, or any T, any *N*, M1a, which typically requires a palliative or individualized treatment approach [4, 10, 11].

Despite the evidence of improved quality of life, lower risk of disease progression, and improved survival benefits, the utilization of guideline‐recommended NAC plus RC in individuals with nonmetastatic MIBC remains low [12]. Delaying RC and the inability to predict responsive tumors are the main reasons for the low uptake of NAC prior to RC [11]. In addition, concerns about increased risk of short‐term post‐surgery outcomes of NAC may explain the low utilization of NAC prior to RC [13, 14]. Information regarding trends in the short‐term post‐surgery outcomes in patients with MIBC who received NAC plus RC is limited.

This study aimed to: 1) characterize the types of treatment received among patients diagnosed with cT2‐T4a, any cN, cM0 MIBC from 2004 to 2017 to determine the proportion of use of RC and trimodal bladder‐sparing treatment over time; 2) describe the utilization of NAC plus RC over time, and the short‐term post‐surgery outcomes, including 30‐day and 90‐day mortality, and readmission, among patients with MIBC diagnosed from 2004 through 2017.

## 2 Materials and Methods

### 2.1 Data Source and Study Population

This study utilizes the National Cancer Database (NCDB), which contains information on over 34 million cancer patients, including about 72.0% of incident malignancies in the United States [15, 16]. It includes data on patients who received an initial cancer diagnosis or the first definitive course of treatment at accredited cancer centers. The study population includes patients with urothelial bladder cancer diagnosed as cT2‐cT4a and any cN and cM0 from 2004 to 2017. We used specific tumor‐node‐metastasis (TNM) staging and tumor histology codes to categorize patients. The NCDB reports major urothelial histology types. Specifically, we focused on transitional cell carcinoma, NOS (histology codes 8120, 8121, 8122, and 8123; constituting 66.7%) and papillary transitional cell carcinoma, NOS (histology codes 8130 and 8131; constituting 33.3%), which together constituted 90.0% of patients with T2‐T4a, any *N*, M0, and were included in this analysis. Additional details on the specific codes and variables utilized to define the study cohort, treatment variables, baseline covariates, and outcomes are available upon request.

### 2.2 Measures

#### 2.2.1 Treatments

We categorized the treatment groups into RC, TURBT, PC, systemic chemotherapy, systemic immunotherapy, radiation therapy, and other treatments. Specific codes of the “surgical procedure performed at the primary site at any facility” variable (i.e., RX_SUMM_SURG_PRIM_SITE variable) [13] were used to identify the type of surgical treatment received by each patient. The RC recipients were further categorized into RC‐only, RC plus NAC, RC plus adjuvant chemotherapy (AC), RC plus NAC and AC, and RC plus other treatments. Patients who received TURBT but no other surgery (e.g., no RC) were further categorized based on the receipt of chemotherapy, radiation therapy, or both. Among patients categorized as receiving TURBT and chemotherapy alone, we examined the timing of TURBT relative to chemotherapy. We excluded patients (*n* = 1,652) who received TURBT and chemotherapy on the same day. We hypothesize that these MIBC patients likely received intravesical chemotherapy rather than systemic chemotherapy, which aligns with real‐world clinical practice.

#### 2.2.2 Outcomes

The study outcomes were: 30‐ and 90‐day post‐surgery mortality, surgical inpatient length of stay, 30‐day readmission, and pathologic response outcomes (i.e., pathologic complete response (pCR), pathologic downstaging (pDS), and other pathologic response). This study focused on short‐term post‐surgical outcomes, such as pCR, due to its clinical relevance and feasibility of assessment within the NCDB. The term pCR specifically applies to patients undergoing RC after NAC, and thus, these outcomes were analyzed in the RC group. Examining outcomes such as complications or survival for the non‐radical cystectomy cohort (e.g., chemoradiotherapy group) presents critical challenges with the NCDB data. Survival analysis to develop treatment effect estimates using the NCDB is subject to immortal time bias, as the timing of treatment initiation often differs significantly between treatment groups [17, 18]. This bias can lead to overestimation of survival estimates for adjuvant therapies, especially when comparing groups with inherently different timelines for treatment initiation (e.g., NAC plus RC versus chemoradiotherapy), potentially resulting in misleading results [17]. As reported in prior research, the NCDB's survival data may not be robust for such comparative analyses [17]. The NCDB does not reliably capture treatment‐related complications following chemoradiotherapy or differentiate between complications directly related to NAC versus radiotherapy [19]. This limits our ability to assess post‐treatment outcomes in the nonsurgical cohort comprehensively, hence the study's decision to examine short‐term post‐surgical outcomes among the RC cohort.

#### 2.2.3 Baseline Characteristics

This study also evaluated baseline patient, tumor, and treating facility characteristics. These included factors such as age, sex, race, ethnicity, health insurance status, Charlson Deyo comorbidity score, tumor stage, lymphovascular invasion, surgical margin status, and facility characteristics such as type, location, and US Census region.

### 2.3 Statistical Analysis

#### 2.3.1 MIBC And Perioperative Treatment Patterns and Trends

We compared baseline demographic, tumor‐level, and treating facility‐level characteristics across treatment groups using the Chi‐square test and Wilcoxon rank sum test for categorical and continuous variables, respectively. We calculated the proportion of patients for each year from 2004 to 2017 who received the MIBC treatments for the total sample and stratified by clinical T staging groups. We conducted a nonparametric Mann‐Kendall trend test [20, 21, 22] and estimated *p*‐value for statistical differences in the monotonic trends in the utilization of each MIBC and RC treatment category year‐over‐year. Similar statistical analyses were conducted, using Cochran–Armitage linear trend test [23] and estimated bootstrap‐adjusted *p*‐value, for the MIBC and RC treatment groups, stratified by three time periods.

#### 2.3.2 Pathologic Response and Postoperative Outcomes

Among MIBC patients who received RC plus NAC, or RC plus NAC and AC, we compared the prevalence of the baseline characteristics across the pathologic response outcomes, using the Chi‐square test. Lastly, for each time period, we estimated the prevalence of 30‐, and 90‐day post‐surgery mortality, and readmissions within 30 days from surgical discharge, for recipients of RC overall, and stratified by NAC status. We conducted a two‐tailed Cochran–Armitage linear trend test and estimated bootstrap‐adjusted *p*‐value for statistical differences in the trends in each post‐surgical outcome category across the three time periods.

All statistical analyses were conducted using SAS Studio (version 9.4, SAS Institute Inc., Cary, NC). All *p*‐values were reported as two‐sided, and *p*‐values less than 0.05 were considered statistically significant.

## 3 Results

**FIGURE 1:**
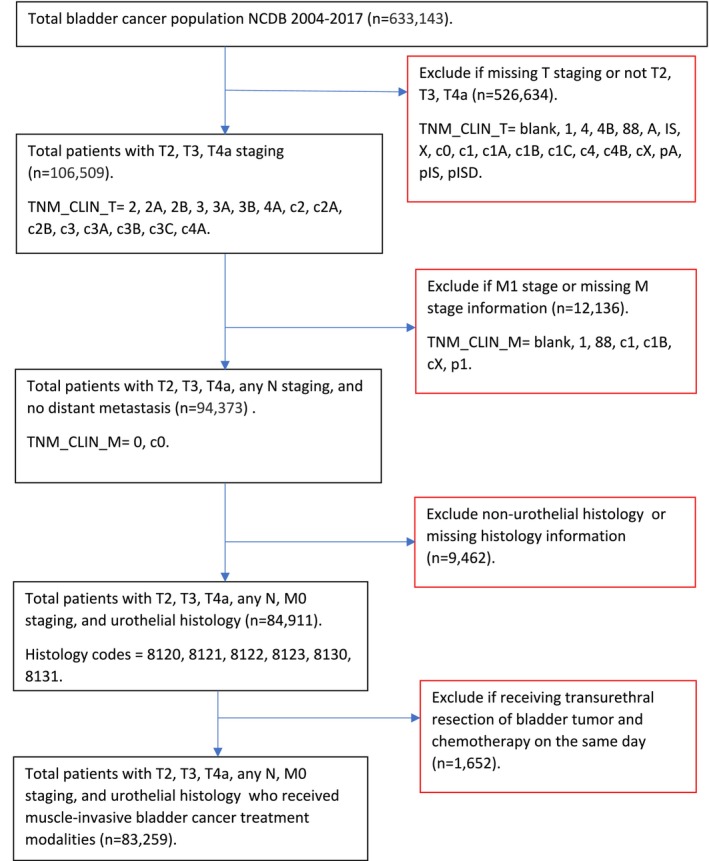
Flow Diagram of muscle‐invasive bladder cancer (MIBC) Cohort Creation.

There were 633,143 individuals diagnosed with bladder cancer in the NCDB from 2004 through 2017. A total of 83,259 met the inclusion criteria (Figure 1). The median (Interquartile range, IQR) age of the analytic cohort was 73 years (17 years), and male patients comprised 74.0% of the sample (Table 1). Various clinical and contextual characteristics were statistically significantly associated with the MIBC treatment utilized. In particular, compared to patients receiving trimodal treatment, those receiving RC were younger (72.1% vs. 40.5% for ages 74 years or younger). Similarly, compared to patients receiving trimodal treatment, MIBC patients who underwent RC had a lower proportion of patients with a Charlson Deyo Comorbidity score of three (2.7% vs. 4.8%). Additionally, MIBC patients who received RC had a lower proportion of patients with residual tumors (10.2% vs. 11.5%) compared to patients receiving trimodal treatment.

**TABLE 1:** Characteristics of patients with MIBC, overall and by treatment receipt, 2004–2017.

	Treatment receipt							
	Overall	RC (w/or w/o NAC/AC)	TURBT + C^a^	TURBT + R	TURBT+C + R	PC	Others^**b**^	*p* ^d^
	*N* (%^**c**^)	*N* (%^**c**^)	*N* (%^**c**^)	*N* (%^**c**^)	*N* (%^**c**^)	*N* (%^**c**^)	*N* (%^**c**^)	
All MIBC patients	83,259 (100)	34,715 (41.7)	6,171 (7.4)	3,783 (4.5)	7,372 (8.9)	1,904 (2.3)	29,314 (35.2)	
Patient‐level								
Median age (IQR), years	73 (17)	69 (14)	70 (15)	83 (10)	77 (14)	74 (16)	78 (16)	
Age groups, years								
< 65	20,988 (25.2)	12,336 (35.5)	2,044 (33.1)	229 (6.1)	1,085 (14.7)	460 (24.2)	4,834 (16.5)	< 0.01
65–74	24,080 (28.9)	12,705 (36.6)	2,065 (33.5)	486 (12.9)	1,901 (25.8)	552 (29.0)	6,371 (21.7)	
75–84	25,526 (30.7)	8,642 (24.9)	1,652 (26.7)	1,543 (40.8)	3,082 (41.8)	641 (33.7)	9,966 (34.0)	
≥ 85	12,665 (15.2)	1,032 (3.0)	410 (6.6)	1,525 (40.3)	1,304 (17.7)	251 (13.2)	8,143 (27.8)	
Sex								
Female	21,747 (26.1)	8,458 (24.4)	1,576 (25.5)	1,218 (32.2)	1,889 (25.6)	486 (25.5)	8,120 (27.7)	< 0.01
Male	61,512 (73.9)	26,257 (75.6)	4,595 (74.5)	2,565 (67.8)	5,483 (74.4)	1,418 (74.5)	21,194 (72.3)	
Race and ethnicity								
Non‐Hispanic White	73,389 (88.2)	31,070 (89.5)	5,244 (85.0)	3,272 (86.5)	6,490 (88)	1,697 (89.1)	25,616 (87.4)	< 0.01
Non‐Hispanic Black	5,300 (6.4)	1,821 (5.3)	514 (8.3)	317 (8.4)	501 (6.8)	107 (5.6)	2,040 (7.0)	
Hispanic	2,268 (2.7)	864 (2.5)	222 (3.6)	95 (2.5)	187 (2.5)	42 (2.2)	858 (2.9)	
Asian + others	2,302 (2.8)	960 (2.8)	191 (3.1)	99 (2.6)	194 (2.6)	58 (3.1)	800 (2.7)	
Primary payer								
Government insurance (Medicaid, Medicare, Other gov't)	60,187 (72.3)	22,416 (64.6)	4,148 (67.2)	3,284 (86.8)	5,963 (80.9)	1,347 (70.8)	23,029 (78.6)	< 0.01
Private insurance	20,169 (24.2)	11,051 (31.8)	1,728 (28.0)	410 (10.8)	1,237 (16.8)	505 (26.5)	5,238 (17.9)	
Uninsured^e^	2,903 (3.5)	1,248 (3.6)	295 (4.8)	89 (2.4)	172 (2.3)	52 (2.7)	1,047 (3.6)	
Charlson Deyo Comorbidity score								
0	55,631 (66.8)	23,839 (68.7)	4,365 (70.7)	2,277 (60.2)	4,763 (64.6)	1,282 (67.3)	19,105 (65.2)	< 0.01
1	18,169 (21.8)	7,610 (21.9)	1,190 (19.3)	891 (23.6)	1,607 (21.8)	428 (22.5)	6,443 (22.0)	
2	6,299 (7.6)	2,333 (6.7)	415 (6.7)	365 (9.7)	651 (8.8)	130 (6.8)	2,405 (8.2)	
3	3,160 (3.8)	933 (2.7)	201 (3.3)	250 (6.6)	351 (4.8)	64 (3.4)	1,361 (4.6)	
Tumor‐level								
AJCC clinical N Stage								
cN0	74,405 (89.4)	31,017 (89.4)	5,009 (81.2)	3,436 (90.8)	6,685 (90.7)	1,766 (92.8)	26,492 (90.4)	< 0.01
cN1	2,572 (3.1)	1,199 (3.5)	397 (6.4)	94 (2.5)	260 (3.5)	37 (1.9)	585 (2.0)	
cN2/cN3	6,202 (7.5)	2,467 (7.1)	757 (12.3)	253 (6.7)	421 (5.7)	100 (5.3)	2,204 (7.5)	
cNX/missing	80 (0.1)	32 (0.1)	*	*	*	*	33 (0.1)	
AJCC clinical T Stage								
cT2	68,831 (82.7)	28,391 (81.8)	4,886 (79.2)	3010 (79.6)	6,155 (83.5)	1,543 (81.0)	24,846 (84.8)	< 0.01
cT3	8,866 (10.7)	4,447 (12.8)	760 (12.3)	480 (12.7)	808 (11)	334 (17.5)	2,037 (7.0)	
cT4a	5,562 (6.7)	1,877 (5.4)	525 (8.5)	293 (7.8)	409 (5.6)	27 (1.4)	2,431 (8.3)	
Lymphovascular invasion								
Not present	23,947 (28.8)	11,631 (33.5)	1,680 (27.2)	809 (21.4)	1,992 (27.0)	619 (32.5)	7,216 (24.6)	< 0.01
Present or identified	13,053 (15.7)	8,377 (24.1)	753 (12.2)	330 (8.7)	747 (10.1)	290 (15.2)	2,556 (8.7)	
Missing or unknown^f^	46,259 (55.6)	14,707 (42.3)	3,738 (60.6)	2,644 (69.9)	4,633 (62.8)	995 (52.3)	19,542 (66.7)	
Surgical margin status								
No residual tumor	38,737 (46.5)	29,306 (84.4)	1,070 (17.3)	561 (14.8)	1,120 (15.2)	1,381 (72.5)	5,299 (18.1)	< 0.01
Residual tumor	8,877 (10.7)	3,545 (10.2)	801 (13.0)	544 (14.4)	850 (11.5)	320 (16.8)	2817 (9.6)	
Unknown or not applicable	35,645 (42.8)	1,864 (5.4)	4,300 (69.7)	2,678 (70.8)	5,402 (73.3)	203 (10.7)	21,198 (72.3)	
Treating facility‐level								
Facility type								
Community	7,931 (9.5)	1887 (5.4)	670 (10.9)	449 (11.9)	842 (11.4)	172 (9.0)	3,911 (13.3)	< 0.01
Comprehensive community	32,829 (39.4)	10,539 (30.4)	2,436 (39.5)	1,841 (48.7)	3,338 (45.3)	848 (44.5)	13,827 (47.2)	
Academic	31,650 (38.0)	18,160 (52.3)	2323 (37.6)	936 (24.7)	2,175 (29.5)	613 (32.2)	7,443 (25.4)	
Integrated cancer network^g^	10,849 (13.0)	4,129 (11.9)	742 (12.0)	557 (14.7)	1,017 (13.8)	271 (14.2)	4,133 (14.1)	
Facility location (2003 urban/rural)								
Metro	65,431 (78.6)	26,435 (76.2)	4,938 (80.0)	3,083 (81.5)	5,903 (80.1)	1,486 (78.1)	23,586 (80.5)	< 0.01
Urban	13,427 (16.1)	6,035 (17.4)	949 (15.4)	573 (15.2)	1,136 (15.4)	321 (16.9)	4,413 (15.1)	
Rural^h^	4401 (5.3)	2,245 (6.5)	284 (4.6)	127 (3.4)	333 (4.5)	97 (5.1)	1,315 (4.5)	
Facility location (2013 urban/rural)								
Metro	66,780 (80.2)	27,046 (77.9)	5,054 (81.9)	3,128 (82.7)	6,026 (81.7)	1,508 (79.2)	24,018 (81.9)	< 0.01
Urban	12,219 (14.7)	5,484 (15.8)	842 (13.6)	535 (14.1)	1,034 (14.0)	301 (15.8)	4,023 (13.7)	
Rural^h^	4,260 (5.1)	2,185 (6.3)	275 (4.5)	120 (3.2)	312 (4.2)	95 (5.0)	1,273 (4.3)	
Facility location (census region)								
Northeast	18,861 (22.7)	7,435 (21.4)	1,440 (23.3)	1,008 (26.7)	1,739 (23.6)	426 (22.4)	6,813 (23.2)	< 0.01
Midwest	33,019 (39.7)	13,355 (38.5)	2,548 (41.3)	1543 (40.8)	3,100 (42.1)	726 (38.1)	11,747 (40.1)	
South	17,925 (21.5)	8,090 (23.3)	1,185 (19.2)	682 (18.0)	1,247 (16.9)	438 (23.0)	6,283 (21.4)	
West^i^	13,454 (16.2)	5,835 (16.8)	998 (16.2)	550 (14.5)	1,286 (17.4)	314 (16.5)	4,471 (15.3)s	
Travel distance to treating facility, miles								
≤ 10	37,386 (44.9)	11,552 (33.3)	2,868 (46.5)	2,196 (58.1)	3,841 (52.1)	914 (48)	16,015 (54.6)	< 0.01
10− < 50	28,402 (34.1)	12,647 (36.4)	2,148 (34.8)	1,225 (32.4)	2,443 (33.1)	595 (31.3)	9,344 (31.9)	
≥ 50	10,616 (12.8)	6,995 (20.2)	593 (9.6)	173 (4.6)	496 (6.7)	243 (12.8)	2,116 (7.2)	
Missing	6,855 (8.2)	3,521 (10.1)	562 (9.1)	189 (5.0)	592 (8.0)	152 (8.0)	1,839 (6.3)	

Abbreviations: AJCC, American Joint Committee on Cancer.C, Chemotherapy; IQR, Interquartile range; MIBC, Muscle‐invasive bladder cancer; *N*, Number; PC, Partial cystectomy; R, Radiotherapy; RC, Radical cystectomy; TURBT, Transurethral resection of bladder tumor.

^a^ The TURBT plus systemic chemotherapy cohort were predominantly (94.5%) MIBC patients who received adjuvant systemic chemotherapy after receiving diagnostic TURBT. This analysis excluded 1652 patients with MIBC who were categorized as having received TURBT and systemic chemotherapy on the same day. We hypothesize that these MIBC patients likely received intravesical chemotherapy rather than systemic chemotherapy, which aligns with real‐world clinical practice.

^b^ Others category consists of patients with MIBC who received TURBT‐only, or who did not meet the bladder sparing, RC, or PC criteria.

^c^ Column percentage.

^d^ Indicate the *p*‐value for statistical difference between the RC route, TURBT + C, TURBT + R, TURBT + C + R, PC, and others categories by the specified demographic‐, tumor‐, or health facility‐level factor.

^e^ Uninsured category includes individuals with missing primary payer at diagnosis; Overall, the proportion of missingness for the primary payer at diagnosis variable is 1.4%.

^f^ The “Missing” and “Unknown” categories have been combined as “Missing or unknown.” The “Unknown” category means it was “unknown if lymph‐vascular invasion is present, or indeterminate.”

^g^ Integrated cancer network category includes individuals with missing facility type; Overall, the proportion of missingness for the facility type variable is less than 1%.

^h^ Rural category includes individuals with missing facility location; Overall, the proportion of missingness for the facility location variable is 3.1%.

^i^ West region category includes individuals with missing facility location; Overall, the proportion of missingness for the facility location variable is < 1%.

* Utilized to denote or mask cell(s) with size(s) less than the reportable value (10) permissible by the NCDB data use agreement [24].

Among those treated with RC, those receiving RC plus NAC were aged 74 years or younger (83.2% vs. 61.1%) compared to recipients of RC‐only (Table 2). Also, 2.2% of RC plus NAC recipients had a Charlson Deyo Comorbidity score of three compared to 3.2% of those who received RC‐only. Furthermore, among RC recipients, those who received NAC prior to RC had a lower proportion of patients with residual tumors (7.0% vs. 9.7%) compared to RC‐only patients.

**TABLE 2:** Characteristics of patients with MIBC who received radical cystectomy, overall and by treatment subgroups, 2004–2017.

	MIBC patients who received RC						*p* ^d^
	Overall^a^	RC‐only	RC + NAC only	RC + AC only	RC + NAC + AC	RC + others^b^	
	*N* (%^c^)	*N* (%^c^)	*N* (%^c^)	*N* (%^c^)	*N* (%^c^)	*N* (%^c^)	
All patients who received RC^e^	34,715 (100)	15,813 (45.6)	7,308 (21.1)	7,456 (21.5)	1,725 (5.0)	1,616 (4.7)	
Patient‐level							
Median age (IQR), years	69 (14)	72 (14)	66 (13)	67 (13)	65 (14)	68 (14)	
Age groups, years							
< 65	12,336 (35.5)	4,285 (27.1)	3,192 (43.7)	3,038 (40.8)	823 (47.7)	593 (36.7)	< 0.01
65–74	12,705 (36.6)	5,369 (34.0)	2,884 (39.5)	2,955 (39.6)	630 (36.5)	592 (36.6)	
75–84	8,642 (24.9)	5,310 (33.6)	1,185 (16.2)	1,383 (18.6)	*	*	
≥ 85	1,032 (3.0)	849 (5.4)	47 (0.6)	80 (1.1)	*	*	
Sex							
Female	8,458 (24.4)	3,905 (24.7)	1744 (23.9)	1,755 (23.5)	400 (23.2)	428 (26.5)	< 0.01
Male	26,257 (75.6)	11,908 (75.3)	5,564 (76.1)	5,701 (76.5)	1,325 (76.8)	1,188 (73.5)	
Race and Ethnicity							
Non‐Hispanic White	31,070 (89.5)	14,156 (89.5)	6,517 (89.2)	6,715 (90.1)	1,582 (91.7)	1,400 (86.6)	
Non‐Hispanic Black	1,821 (5.3)	832 (5.3)	389 (5.3)	350 (4.7)	80 (4.6)	123 (7.1)	
Hispanic	864 (5.3)	389 (2.46)	194 (2.65)	184 (2.47)	28 (1.62)	45 (2.78)	
Asian + others	960 (2.8)	436 (2.8)	208 (2.9)	207 (2.8)	35 (2.0)	48 (3.0)	
Primary payer							
Government insurance (Medicaid, Medicare, Other gov't)	22,416 (64.6)	11,184 (70.7)	4245 (58.1)	4,532 (60.8)	989 (57.3)	1054 (65.2)	< 0.01
Private insurance	11,051 (31.8)	4071 (25.7)	2,799 (38.3)	2,656 (35.6)	680 (39.4)	492 (30.5)	
Uninsured^f^	1,248 (3.6)	558 (3.5)	264 (3.6)	268 (3.6)	56 (3.3)	70 (4.3)	
Charlson/Deyo Comorbidity score							
0	23,839 (68.7)	10,308 (65.2)	5,321 (72.8)	5221 (70.0)	1,266 (73.4)	1,129 (69.9)	< 0.01
1	7,610 (21.9)	3720 (23.5)	1,436 (19.7)	1640 (22.0)	313 (18.1)	334 (20.7)	
2	2,333 (21.9)	1277 (8.1)	390 (5.3)	432 (5.8)	105 (6.1)	103 (6.7)	
3	933 (2.7)	508 (3.2)	161 (2.2)	163 (2.2)	41 (2.4)	50 (3.1)	
Tumor‐level							
AJCC clinical N Stage							
cN0	31,017 (89.4)	14,557 (92.1)	6459 (88.4)	6503 (87.2)	1517 (87.9)	1,347 (83.4)	< 0.01
cN1	1199 (89.4)	279 (1.8)	369 (5.1)	332 (4.5)	77 (4.5)	100 (6.2)	
cN2/cN3	2,467 (7.1)	964 (6.1)	476 (6.5)	611 (8.2)	*	*	
cNX/missing	32 (0.1)	*	*	*	*	*	
AJCC clinical T Stage							
cT2	28,391 (81.8)	13,358 (84.5)	5,862 (80.2)	6,022 (80.8)	1425 (82.6)	1,157 (71.6)	< 0.01
cT3	4,447 (12.8)	1,787 (11.3)	1014 (13.9)	1003 (13.5)	200 (11.6)	266 (16.5)	
cT4a	1,877 (12.8)	668 (4.2)	432 (5.9)	431 (5.8)	100 (5.8)	193 (11.9)	
Lymphovascular invasion							
Not present	11,631 (33.5)	4,832 (30.6)	3239 (44.3)	2,408 (32.3)	701 (40.6)	401 (24.8)	< 0.01
Present or identified	8,377 (33.5)	3,258 (20.6)	1778 (24.3)	2309 (31.0)	478 (27.7)	533 (33)	
Missing or unknown^g^	14,707 (42.3)	7,723 (48.9)	2,291 (31.3)	2,739 (36.8)	546 (31.7)	682 (42.2)	
Surgical margin status							
No residual tumor	29,306 (84.4)	13,555 (85.7)	6,507 (89)	6,028 (80.9)	1,474 (85.5)	1,080 (66.8)	< 0.01
Residual tumor	3,545 (10.2)	1,538 (9.7)	513 (7.0)	846 (11.4)	140 (8.1)	428 (26.5)	
Unknown or not applicable	1,864 (10.2)	720 (4.6)	288 (3.9)	582 (7.8)	111 (6.4)	108 (6.7)	
Treating facility‐level							
Facility type							
Community	1,887 (5.4)	861 (5.4)	324 (4.4)	457 (6.1)	85 (4.9)	127 (7.9)	< 0.01
Comprehensive community	10,539 (30.4)	5,135 (32.5)	1,725 (23.6)	2,353 (31.6)	552 (32.0)	516 (31.9)	
Academic	18,160 (52.3)	8,029 (50.8)	4,343 (59.4)	3,721 (49.9)	876 (50.8)	778 (48.1)	
Integrated cancer network^h^	4,129 (11.9)	1,788 (11.3)	916 (12.5)	925 (12.4)	212 (12.3)	195 (12.1)	
Facility location (2003 urban/rural)							
Metro	26,435 (76.2)	11,956 (75.6)	5,613 (76.8)	5689 (76.3)	1,294 (75)	1,270 (78.6)	0.02
Urban	6035 (6.5)	2798 (17.7)	1,240 (17.0)	1,308 (17.5)	316 (18.3)	256 (15.8)	
Rural	2,245 (6.5)	1,059 (6.7)	455 (6.2)	459 (6.2)	115 (6.7)	90 (5.6)	
Facility location (2013 urban/rural)							
Metro	27,046 (77.9)	12,231 (77.4)	5,741 (78.6)	5,822 (78.1)	1,316 (76.3)	1,311 (81.1)	< 0.01
Urban	5,484 (6.3)	2,552 (16.1)	1,123 (15.4)	1,185 (15.9)	295 (17.1)	221 (13.7)	
Rural	2,185 (6.3)	1,030 (6.5)	444 (6.1)	449 (6.0)	114 (6.6)	84 (5.2)	
Facility location (census region)							
Northeast	7,435 (21.4)	3,361 (21.3)	1,541 (21.1)	1,640 (22.0)	354 (20.5)	364 (22.5)	< 0.01
Midwest	13,355 (38.5)	5950 (37.6)	3,016 (41.3)	2791 (37.4)	660 (38.3)	660 (40.8)	
South	8,090 (21.4)	3,813 (24.1)	1,651 (22.6)	1,740 (23.3)	367 (21.3)	336 (20.8)	
West^i^	5,835 (16.8)	2,689 (17)	1,100 (15.1)	1,285 (17.2)	344 (19.9)	256 (15.8)	
Travel distance to treating facility, miles							
≤ 10	11,552 (33.3)	5,578 (35.3)	2016 (27.6)	2,537 (34.0)	519 (30.1)	611 (37.8)	< 0.01
10− < 50	12,647 (33.3)	5,736 (36.3)	2,695 (36.9)	2,722 (36.5)	626 (36.3)	568 (35.2)	
≥ 50	6,995 (20.2)	3,330 (21.1)	1,612 (22.1)	1,291 (17.3)	330 (19.1)	286 (17.7)	
Missing	3,521 (10.1)	1,169 (7.4)	985 (13.5)	906 (12.2)	250 (14.5)	151 (9.3)	

Abbreviations: AC, adjuvant chemotherapy; AJCC, American Joint Committee on Cancer; C, chemotherapy; IQR, interquartile range; MIBC, muscle‐invasive bladder cancer; NAC, neoadjuvant chemotherapy; *N*, number; RC, radical cystectomy.

^a^ Overall—Includes information on MIBC patients who received chemotherapy, however, the chemotherapy sequence relative to RC is unknown or missing (*n* = 797 constituting 2.3% of recipients of RC).

^b^ RC + other category consists of recipients of RC who received radiotherapy or immunotherapy in addition to chemotherapy, or RC plus immunotherapy only.

^c^ Column percentage.

^d^ Indicate the *p*‐value for statistical difference between the RC treatment categories by the specified demographic‐, tumor‐, or treating facility‐level characteristics.

^e^ Row percentage; The row percentages of RC‐only, NAC, AC, NAC + AC, RC + others, and the excluded RC with the unknown chemotherapy sequence, sum up to 100% for the RC treatment category.

^f^ Uninsured category includes individuals with missing primary payer at diagnosis; Overall, the proportion of missingness for the primary payer at diagnosis variable is 1.3%.

^g^ The “Missing” and “Unknown” categories have been combined as “Missing or unknown.” The “Unknown” category means it was “unknown if lymph‐vascular invasion is present, or indeterminate.”

^h^ Integrated cancer network category includes individuals with missing facility type; Overall, the proportion of missingness for the facility type variable is < 1.0%.

^i^ West region category includes individuals with missing facility location; Overall, the proportion of missingness for the facility location variable is less than 1.0%.

* Utilized to denote or mask cell(s) with size(s) less than the reportable value (10) permissible by the NCDB data use agreement [24].

### 3.1 MIBC Treatment Patterns and Trends

**FIGURE 2:**
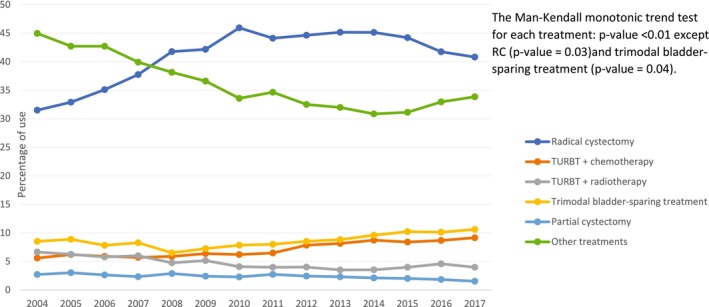
Trends in MIBC treatment patterns, overall, 2004–2017. MIBC, Muscle‐invasive bladder cancer; PC, Partial cystectomy; RC, Radical cystectomy; TURBT, Transurethral resection of bladder tumor.

**FIGURE 3:**
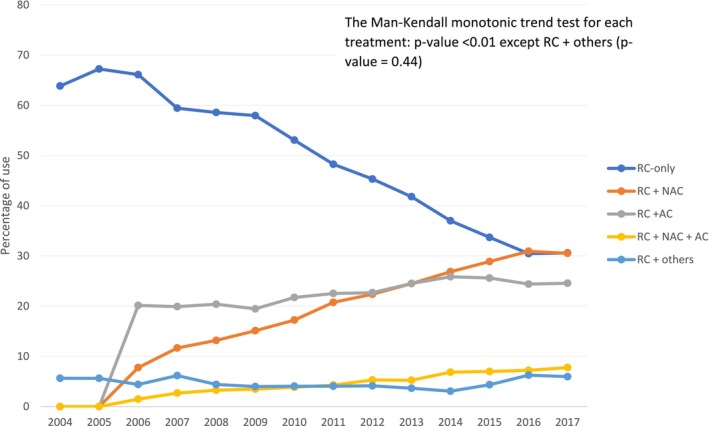
Trends in perioperative treatment patterns in MIBC patients who received radical cystectomy, 2004–2017. AC, Adjuvant chemotherapy; MIBC, Muscle‐invasive bladder cancer; NAC, Neoadjuvant chemotherapy; RC, Radical cystectomy.

Overall, of the 83,259 individuals included, those who received RC, trimodal bladder‐sparing treatment, and TURBT plus chemotherapy were 34,715 (41.7%), 7,372 (8.9%), and 6,171 (7.4%), respectively (Table 1). A substantial proportion (29,314; 35.2%) of MIBC patients received “other treatments,” which included TURBT‐only, or treatments that did not meet the bladder sparing, PC, or RC criteria. The proportion of MIBC patients who utilized guideline‐recommended treatments, whether RC or trimodal bladder‐sparing treatment, increased significantly (i.e., *p* < 0.05) over the study time period, as reported in Figures 2 and 3. The use of TURBT plus systemic chemotherapy, a potentially less effective bladder‐sparing treatment approach for MIBC [25], increased significantly over the study period, particularly from 2010 through 2017. From the early (2004–2008) to the late (2014–2017) time period, the prevalence of use of: (1) RC increased significantly from 7,791 (36.4%) to 13,856 (42.8%); (2) trimodal bladder‐sparing treatment increased significantly from 1,687 (7.9%) to 3,300 (10.2%); and (3) TURBT plus chemotherapy increased significantly from 1,255 (5.9%) to 2,840 (8.8%). Results for RC and trimodal bladder‐sparing treatment were similar when stratified by clinical T staging at diagnosis.

### 3.2 Perioperative Treatment Patterns and Trends

Among the 34,715 who received RC, the median (IQR) age was 69 (14) years, with males constituting 75.6% of the RC cohort. Of the RC cohort, 7,308 (21.0%), 15,813 (46.0%), and 7,456 (21.0%) received NAC (i.e., NAC plus RC only), RC‐only, and AC (i.e., RC plus AC only), respectively (Table 2). Demographic, tumor‐level, and facility‐level factors were associated with the RC treatment receipt in bivariate analyses.

Table 3 shows the trends in perioperative treatment patterns in MIBC patients who received RC. The prevalence of NAC use increased: 1) from 609 (7.8%) to 4,078 (29.4%; *p* < 0.01) overall; and 2) from 430 (7.1%) to 3,390 (28.9%; *p* < 0.01) in patients with cT2 stage MIBC, from the early to the late time period. Conversely, the proportion of RC recipients who received RC‐only decreased over time (*p* < 0.01) (Table 3 and Figure 3).

**TABLE 3:** Trends in perioperative treatment patterns in MIBC patients who received radical cystectomy, overall and by clinical staging, 2004–2017.

	Year of diagnosis for patients with MIBC receiving RC treatment				*p* ^b^
	Overall	2004–2008	2009–2013	2014–2017	
	*N* (%^a^)	*N* (%^a^)	*N* (%^a^)	*N* (%^a^)	
Treatment pattern by clinical T staging					
*RC overall*	34,715*	7,791	13,068	13,856	< 0.01
RC only	15,813 (45.6)	4,850 (62.3)	6,428 (49.2)	4,535 (32.7)	< 0.01
RC + NAC	7,308 (21.1)	609 (7.8)	2,621 (20.1)	4,078 (29.4)	< 0.01
RC + AC	7,456 (21.5)	1,082 (13.9)	2,902 (22.2)	3,472 (25.1)	< 0.01
RC + NAC + AC	1,725 (5.0)	142 (1.8)	581 (4.5)	1,002 (7.2)	< 0.01
RC + other^c^	1,616 (4.7)	402 (5.2)	519 (4.0)	695 (5.0)	1.00
*cT2*	28,391	6,088	10,588	11,715	< 0.01
RC only	13,358 (47.1)	3,976 (65.3)	5,405 (51.1)	3,977 (34.0)	< 0.01
RC + NAC	5,862 (20.7)	430 (7.1)	2,042 (19.3)	3,390 (28.9)	< 0.01
RC + AC	6,022 (21.2)	808 (13.3)	2,301 (21.7)	2,913 (24.9)	< 0.01
RC + NAC + AC	1,425 (5.0)	108 (1.8)	465 (4.4)	852 (7.3)	< 0.01
RC + other^c^	1,157 (4.1)	277 (4.6)	362 (3.4)	518 (4.4)	1.00
*cT3/cT4a*	6,324	1,703	2,480	2,141	< 0.01
RC only	2,455 (38.8.2)	874 (51.3)	1,023 (41.3)	558 (26.1)	< 0.01
RC + NAC	1,446 (22.9)	179 (10.5)	579 (23.3)	688 (32.1)	< 0.01
RC + AC	1,434 (22.7)	274 (16.1)	601 (24.2)	559 (26.1)	< 0.01
RC + NAC + AC	300 (4.7)	34 (2.0)	116 (4.7)	150 (7.0)	< 0.01
RC + other^c^	459 (7.3)	125 (7.3)	157 (6.3)	177 (8.3)	0.72

Abbreviations: AC, adjuvant chemotherapy; MIBC, muscle‐invasive bladder cancer; *N*, number; NAC, neoadjuvant chemotherapy; RC, radical cystectomy.

* RC + C subcategories (RC + NAC, RC + NAC + AC, and RC + AC) presented in the table exclude information on MIBC patients who received chemotherapy and whose chemotherapy sequence relative to RC is unknown or missing (*n* = 797).

^a^ Column percentage.

^b^ Indicate the bootstrap‐adjusted *p*‐value for statistical difference in the trends in the utilization of each RC treatment category across the three time periods. This was estimated using the two‐tailed Cochran‐Armitage linear trend test.

^c^ RC + other category consists of recipients of RC who received radiotherapy or immunotherapy in addition to chemotherapy.

### 3.3 Pathologic Response Outcomes Among NAC Recipients

Among 7,828 MIBC patients who received NAC prior to RC, 1,498 achieved pCR (19.1%) and 1,270 (16.2%) achieved pDS (Table 4). Additionally, tumor‐ and facility‐level variables were associated with the surgical pathologic response outcomes (Table 4). At the time of diagnosis, pathological factors associated with pCR included a lower prevalence of clinical stages and lymphovascular invasion (LVI) (3.2% vs. 8.4% for N2/N3, 3.1% vs. 6.8% for T4a, and 11.4% vs. 35.1% for LVI, all with *p* < 0.01).

**TABLE 4:** Characteristics and surgical pathologic outcomes of patients MIBC (cT2, cT3, cT4a, any cN, cM0) who received neoadjuvant chemotherapy and radical cystectomy, 2004–2017.

	Patients who achieved pCR	Patients who achieved pDS but not pCR	Patients who did not achieve pCR or pDS^a^	*p*
	*N* (%^b^)	*N* (%^b^)	*N* (%^b^)	
Overall (*N* = 7,828**)	1,498 (100^c^)	1,270 (100^c^)	5,060 (100Th^c^)	
Patient‐level				
Median age at diagnosis (IQR), years	65 (14)	66 (13)	66 (13)	
Age groups, years				
< 65	691 (46.1)	573 (45.1)	2,195 (43.4)	0.08
65–74	590 (39.4)	501 (39.5)	1,972 (39.0)	
≥ 75^d^	217 (14.5)	196 (15.4)	893 (17.6)	
Sex				
Female	319 (21.3)	255 (20.1)	1,256 (24.8)	< 0.01
Male	1,179 (78.7)	1,015 (79.9)	3,804 (75.2)	
Race				
Non‐Hispanic White	1356 (90.5)	1,152 (90.7)	4,509 (89.1)	0.12
Non‐Hispanic Black	64 (4.3)	53 (4.2)	276 (5.5)	
Hispanic	37 (2.5)	24 (1.9)	140 (2.8)	
Asian + others	41 (2.7)	41 (3.2)	135 (2.7)	
Primary payer at diagnosis				
Government insurance (Medicaid, Medicare, Other gov't)	813 (54.3)	713 (56.1)	3,046 (60.2)	< 0.01
Private insurance	629 (42)	522 (41.1)	1,838 (36.3)	
Uninsured^e^	56 (3.7)	35 (2.8)	176 (3.5)	
Tumor‐level				
*AJCC clinical N Stage*				
cN0	1,394 (93.1)	1,177 (92.7)	4,341 (85.8)	< 0.01
cN1	56 (3.7)	31 (2.4)	294 (5.8)	
cN2/cN3^f^	48 (3.2)	62 (4.9)	425 (8.4)	
AJCC clinical T Stage				
cT2	1,311 (87.5)	1,079 (85)	3,930 (77.7)	< 0.01
cT3	140 (9.4)	141 (11.1)	785 (15.5)	
cT4a	47 (3.1)	50 (3.9)	345 (6.8)	
Lymphovascular invasion				
Not present	773 (51.6)	771 (60.7)	2,034 (40.2)	< 0.01
Present or identified	171 (11.4)	168 (13.2)	1,778 (35.1)	
Missing or unknown^g^	554 (37.2)	331 (26.1)	1,248 (24.7)	
Surgical margin status				
No residual tumor	1,448 (96.7)	1,186 (93.4)	4,383 (86.6)	< 0.01
Residual tumor^h^	*	*	498 (9.8)	
Unknown or not applicable	40 (2.7)	35 (2.8)	179 (3.5)	
Treating facility‐level				
*Facility type*				
Community	52 (3.5)	39 (3.1)	229 (4.5)	< 0.01
Comprehensive community	343 (22.9)	270 (21.3)	1,283 (25.4)	
Academic	915 (61.1)	783 (61.7)	2,948 (58.3)	
Integrated cancer network^i^	188 (12.6)	178 (14.0)	600 (11.9)	
Facility location (2003 urban/rural)				
Metro	1,137 (75.9)	976 (76.9)	3,843 (76.0)	0.38
Urban	251 (16.8)	210 (16.5)	905 (17.9)	
Rural	110 (7.3)	84 (6.6)	312 (6.2)	
Facility location (2013 urban/rural)				
Metro	1,160 (77.4)	1,004 (79.1)	3,924 (77.6)	0.17
Urban	229 (15.3)	183 (14.4)	832 (16.4)	
Rural	109 (7.3)	83 (6.5)	304 (6)	
Facility location (census region)				
Northeast	328 (21.9)	289 (22.8)	1,059 (20.9)	0.07
Midwest	585 (39.1)	525 (41.3)	2,021 (39.9)	
South	358 (23.9)	283 (22.3)	1,127 (22.3)	
West^j^	227 (15.2)	173 (13.6)	853 (16.9)	
Travel distance to treating facility, miles				
≤ 10	389 (26.0)	325 (25.6)	1,427 (28.2)	0.01
10− < 50	500 (33.4)	469 (36.9)	1,910 (37.8)	
≥ 50	344 (23.0)	279 (22.0)	1,099 (21.7)	
Missing	265 (17.7)	197 (15.5)	624 (12.3)	

Abbreviations: AC, adjuvant chemotherapy; AJCC, American Joint Committee on Cancer; C, chemotherapy; pCR, pathologic complete response; pDS, pathologic downstaging; IQR, interquartile range; *N*, Number.

^a^ This category includes individual MIBC (cT2, cT3, cT4a, any cN, cM0) who received RC and NAC and who did not achieve pCR or pDS. Thus, total *N* = pCR + pDS + other, as such, it should not be interpreted as an exposure variable.

^b^ Column percentage.

^c^ The sum total of the column percentages.

^d^ The 75–84 and ≥ 85‐year categories were combined to mask small cell size values; Overall, the proportion of individuals ≥ 85 years is < 1.0%.

^e^ Uninsured category includes individuals with missing primary payer at diagnosis information; Overall, the proportion of missingness for the primary payer at diagnosis variable is 1.3%.

^f^ cN2/cN3 category includes individuals with missing AJCC clinical N staging information; Overall, the proportions of cN2/cN3, and missingness for the AJCC clinical N staging variable are 7.4% and 0.1%, respectively.

^g^ The “Missing” and “Unknown” categories have been combined as “Missing or unknown.” The “Unknown” category means it was “unknown if lymph‐vascular invasion is present, or indeterminate.”

^h^ The residual tumor and margin not evaluable categories were combined to mask small cell size values; Overall, the proportion of individuals with tumor margins not evaluable is 1.6%.

^i^ Integrated cancer network category includes individuals with missing facility type information; Overall, the proportion of missingness for the facility type variable is < 1.0%.

^j^ West category includes individuals with missing facility location information; Overall, the proportion of missingness for the facility location variable is < 1.0%.

* Utilized to denote or mask cell(s) with size(s) less than the reportable value (10) permissible by the NCDB data use agreement [24].

** The pathologic response outcome algorithm for MIBC patients, who received radical cystectomy, was defined using the TNM pathologic T and TNM pathologic *N* variables only. We did not include the TNM pathologic M variable in establishing the pathologic response outcomes. We excluded, from the pathologic response outcomes analysis, a total of 6,074 (17.5%) MIBC patients who received radical cystectomy and had missing or unknown values for TNM pathologic T and TNM pathologic *N* variables.

### 3.4 Trends in Postoperative Outcomes

A total of 30,955 patients with MIBC received RC and had non‐missing post‐surgery outcomes at the time of this analysis (Table 5). Of these, 7,593 (24.5%) received NAC prior to RC. In patients who received NAC relative to those who did not receive NAC, we observed lower 30‐ and 90‐day surgical mortality rates (1.3% vs. 2.5% and 3.8% vs. 6.6%, respectively). Additionally, among RC recipients, we observed a statistically significant and monotonic decrease in the 30‐ and 90‐day post‐surgery mortality rate over the study period (Table 5). The proportion of unplanned readmissions remained statistically unchanged from the early to the late time period (7.4%–7.7%; *p* = 0.88). Among NAC recipients, even though the 30‐day post‐surgery mortality rate decreased from 2.1% in the early time period to 1.4% in the late time period, this change was not monotonic over the time period (*p* = 0.98). Similarly, there were no statistically significant changes in the proportion of unplanned readmissions between the early and late time period (42 [5.6%] vs. 264 [i.e., 7.3%]; *p* = 0.98).

**TABLE 5:** Trends in post‐surgery outcomes in patients with MIBC who received radical cystectomy, overall and by neoadjuvant chemotherapy status, 2004–2016.

	MIBC Patients with RC				*p* ^b^
	2004–2016	2004–2008	2009–2013	2014–2016	
	*N* (%^a^)	*N* (%^a^)	*N* (%^a^)	*N* (%^a^)	
Clinical outcomes					
All patients with RC^*^	30,955	7,791	13,068	10,096	
Mortality					
Died ≤ 30 days post‐surgery	663 (2.1)	218 (2.8)	261 (2.0)	184 (1.8)	< 0.01
Died ≤ 90 days post‐surgery	1,816 (5.9)	555 (7.1)	726 (5.6)	535 (5.3)	< 0.01
Median surgical inpatient stay (IQR), days	7 (4.0)	8 (5.0)	7 (4.0)	7 (4.0)	
Readmission within 30 days from surgical discharge					
No readmission within 30 days from surgical discharge or no surgery at primary site	27,329 (88.3)	6,738 (86.5)	11,553 (88.4)	9,038 (89.5)	< 0.01
Unplanned readmission	2,384 (7.7)	573 (7.4)	1,036 (7.9)	775 (7.7)	0.88
Any planned readmission	582 (1.9)	241 (3.1)	242 (1.9)	99 (1.0)	< 0.01
Unknown	660 (2.1)	239 (3.1)	237 (1.8)	184 (1.8)	< 0.01
Patients with neoadjuvant chemotherapy	7,593	751	3,202	3,640	
Mortality					
Died ≤ 30 days post‐surgery	98 (1.3)	16 (2.1)	30 (0.9)	52 (1.4)	0.98
Died ≤ 90 days post‐surgery	290 (3.8)	35 (4.7)	118 (3.7)	137 (3.8)	0.71
Median surgical inpatient stay (IQR), days	7 (4.0)	8 (5.0)	7 (3.0)	6 (3.0)	
Readmission within 30 days from surgical discharge					
No readmission within 30 days from surgical discharge or no surgery at primary site	6,774 (89.2)	660 (87.9)	2,835 (88.5)	3,279 (90.1)	0.07
Unplanned readmission	570 (7.5)	42 (5.6)	264 (8.2)	264 (7.3)	0.98
Any planned readmission	73 (1.0)	15 (2.0)	37 (1.2)	21 (0.5)	< 0.01
Unknown	176 (2.3)	34 (4.5)	66 (2.1)	76 (2.1)	0.01
Patients without any neoadjuvant chemotherapy^c^	21,194	5,932	9330	5,932	
Mortality					
Died ≤ 30 days post‐surgery	529 (2.5)	186 (3.1)	217 (2.3)	126 (2.1)	< 0.01
Died ≤ 90 days post‐surgery	1,402 (6.6)	466 (7.9)	569 (6.1)	367 (6.2)	< 0.01
Median surgical inpatient stay (IQR), days	7 (4.0)	8 (5.0)	7 (4.0)	7 (4.0)	
Readmission within 30 days from surgical discharge					
No readmission within 30 days from surgical discharge or no surgery at primary site	18,645 (88.0)	5134 (86.6)	8238 (88.3)	5,273 (88.9)	< 0.01
Unplanned readmission	1,696 (8.0)	463 (7.8)	743 (8.0)	490 (8.3)	0.76
Any planned readmission	452 (2.1)	186 (3.1)	192 (2.0)	74 (1.2)	< 0.01
Unknown	401 (1.9)	149 (2.5)	157 (1.7)	95 (1.6)	< 0.01

Abbreviations: MIBC, muscle‐invasive bladder cancer; *N*, number; RC, radical cystectomy.

^a^ Column percentage.

^b^ Indicate the bootstrap‐adjusted *p*‐value for statistical difference in the trends in each post‐surgical outcome category across the three time periods. This was estimated using the two‐tailed Cochran‐Armitage linear trend test.

^c^ The patients without any neoadjuvant category exclude recipients of RC who received radiotherapy or immunotherapy in addition to chemotherapy, RC plus immunotherapy only, or RC + chemotherapy but the chemotherapy sequence is reported missing. The patients whose information was excluded are 2,168.

* Information on recipients of RC (*n* = 3,760) whose year of MIBC diagnosis was 2017 was excluded from the outcomes analyses.

## 4 Discussion

This analysis of the NCDB from 2004 to 2017 shows significantly increased utilization of trimodal bladder‐sparing treatment, TURBT plus chemotherapy, and RC among patients diagnosed with MIBC. Compared to RC, the increased use of trimodal bladder‐sparing treatment from 8.5% to 10.6% appears to be driven by increasing age, increasing comorbidity, or stage cT2 disease. RC remains the standard curative treatment for MIBC. However, not all MIBC patients are suitable candidates for RC. Older age, increased comorbidity burden, or contraindications to major surgery may preclude RC [26]. For these patients, trimodal bladder‐sparing treatment represents an effective and NCCN‐recommended alternative. Beyond its suitability for MIBC patients with significant comorbidities or advanced age, trimodal bladder‐sparing treatment is also a preferred option for MIBC patients who prioritize bladder preservation or decline cystectomy [26, 27]. Trimodal bladder‐sparing treatment is increasingly recognized as a viable option to preserve bladder function while maintaining acceptable survival rates for selected MIBC patients [27]. These factors, combined with advances in radiotherapy and systemic therapies, including immune checkpoint inhibitors [27], may have contributed to the increasing utilization of trimodal bladder‐sparing treatment in the evolving treatment landscape for MIBC. Using the NCDB from 2004 to 2013, Patel and colleagues reported an association between older age, increasing comorbidity, and receiving bladder‐sparing treatment in patients with MIBC [28]. However, the Patel et al. study used a multivariable multinomial logistic regression model to estimate the risk factors for the receipt of bladder‐sparing treatment. As such, their findings do not directly compare to our findings.

Alongside the significant increase in trimodal bladder‐sparing treatment use, we observed a significant rise in the adoption of TURBT plus systemic chemotherapy over the examined period, despite the AUA and NCCN guidelines recommending trimodal bladder‐sparing treatment and NAC before RC for treating MIBC [2, 3, 5]. This TURBT plus systemic chemotherapy cohort was predominantly (94.5%) MIBC patients who received adjuvant systemic chemotherapy after receiving diagnostic TURBT. The finding of increasing use of TURBT plus chemotherapy, a potentially less effective bladder‐sparing treatment [25], warrants further investigation. Based on available studies, the factors contributing to this trend may include variations in physician adherence to treatment guidelines, patient comorbidities that preclude the use of more aggressive treatments, differences in institutional capacities such as availability of specialized surgical teams or equipment, and the potential influence of recent clinical studies suggesting potential benefits of TURBT plus chemotherapy [25, 29, 30, 31, 32]. The increased utilization of TURBT plus chemotherapy between 2010 and 2017 could be attributed to several factors. During the 2010 to 2017 period, there was an expansion of comparative effectiveness research, funded by the Patient‐Centered Outcomes Research Institute (PCORI), highlighting the effectiveness of systemic chemotherapy in various settings, including MIBC treatment with TURBT plus chemotherapy [33]. This evidence may have influenced clinical practice. Additional research will be needed to identify the contributing factors (considering patient‐level, physician‐level, and contextual factors) and the implications for MIBC treatment outcomes.

The present study highlights changes over time in RC treatment patterns and outcomes for patients with MIBC. We observed that the sample of patients undergoing RC was more likely to have a lower proportion of patients with residual tumor compared to the sample of patients treated with trimodal therapy. Similarly, the sample of patients who received NAC in combination with RC had the lowest proportion of patients with residual tumor compared to the sample with RC‐only patients. Residual tumor presence is a critical prognostic factor in MIBC management, with prior studies emphasizing the importance of surgical margin status and location in patient outcomes [34, 35]. Our findings highlight the role of NAC in reducing residual disease burden and improving surgical outcomes. Further investigation into the relationship between surgical margin status and location, as well as its implications for long‐term outcomes, is warranted.

NAC followed by RC remains the standard of care for MIBC. Our study showed a striking increase (7.8% to 29.4%) in the utilization of NAC from the 2004 to 2008 period through the 2013 to 2017 period. NAC before RC has shown efficacy in downstaging tumors, making them more operable [12]. The combination of NAC plus RC has demonstrated superior survival rates compared to RC alone, influencing the growing trends in their utilization for MIBC patients. The observed increased trend in NAC utilization appears to be driven by the increased NAC use in patients with younger age, lower comorbidity, clinical stage cT2 disease (i.e., from 7.1% to 28.9%), who access care at academic hospitals or reside in the Midwest of the US [36]. These findings are consistent with the determinants of NAC use reported by Carvalho et al. using the NCDB from 2004 to 2014 [12, 36].

The utilization of NAC remained low (i.e., < 30%) among MIBC patients across all three time periods. Our findings are consistent with that of Carvalho and colleagues who found NAC use in less than 20.0% of patients with MIBC [12, 36]. We found a significant decrease (i.e., 62.3% to 32.7%) in the use of RC‐only in MIBC patients over the same time frame, highlighting the results of efforts to improve the use of guideline‐recommended treatment for MIBC. Notable efforts to increase utilization of NAC before RC were implemented at both clinician and patient levels. At the clinician level, educational initiatives and clinical guidelines, such as those published by NCCN, AUA, and the American Society of Clinical Oncology (ASCO) [37], were developed to inform clinicians about the benefits of NAC. The establishment of multidisciplinary tumor boards [38], which included various specialists, ensured comprehensive and collaborative treatment planning, incorporating NAC when clinically indicated, and ensuring adherence to guideline‐recommended treatments. In addition, the introduction of Enhanced Recovery After Surgery (ERAS) protocols provided a standardized evidence‐based approach to perioperative care, potentially leading to a more consistent utilization of NAC plus RC [39].

At the patient level, the ERAS protocols involved preoperative counseling and education, including nutritional optimization, and physical conditioning, helping patients better understand their treatment and recovery process, potentially increasing their willingness to undergo NAC plus RC [39]. Additionally, extensive campaigns were launched to raise awareness among MIBC patients about the benefits of NAC [14, 40]. These campaigns, including information shared during clinical consultations and through patient education materials, provided patients with comprehensive information about the potential benefits of NAC and other treatment options [41]. Despite these efforts, the fact that over a third of MIBC patients received RC‐only over time underscores an unmet need in MIBC treatment [23, 25], warranting further research to better understand the contributing factors.

Among RC recipients, the significant steady decrease in post‐surgery mortality from 2004 to 2008 period through the 2013 to 2016 period, may be due to advancements in surgical techniques, improved perioperative care, increased utilization of more effective chemotherapies (i.e., NAC), or better patient selections for treatment [14, 42, 43]. However, our observed decreasing trend in post‐surgery mortality in NAC recipients was not statistically significant and this may be due to the small sample. Across all three time periods, individuals who received any NAC constituted between 9.6% and 36.6%. Aside from the small sample size, there may be variations in the patient characteristics of NAC recipients over time making it challenging to establish significant trends in post‐surgery mortality outcomes. Additionally, the advancements in RC technique, including the use of robotic RC [44], overtime masking the effect of NAC on mortality outcomes, heterogeneity in NAC response, or changes in NAC treatment protocols may have contributed to the difficulty in establishing statistical significance effects [45]. Prior research in individuals aged 70 years and older reported similar clinically acceptable 30‐day post‐surgery mortality rate (i.e., ≤ 2.0%) [42, 46] among NAC users, with statistical significance [14]. However, our observed decline in 30‐day mortality was statistically nonsignificant, necessitating further research with sufficient statistical power.

Our findings suggest a shift in treatment approaches for MIBC, with increasing utilization of NAC and a decrease in the prevalence of RC‐only treatment. In addition, we observed an increasing utilization of chemoradiotherapy, reflecting a growing role or acceptance of bladder‐preserving therapeutic approaches in appropriately selected MIBC patients. The decrease in post‐surgery mortality among RC recipients among NAC users indicates improved outcomes, potentially due to advancement in surgical techniques, improved perioperative care, utilization of more effective chemotherapies, or better patient selections for treatment [14, 40, 41, 43, 47].

This study fills an important gap in evidence regarding the evolving MIBC treatment landscape and trends in health outcomes including post‐surgery mortality and readmission following surgical discharge. These findings support the critical need to identify drivers of the increased use of TURBT plus chemotherapy, and the persistent underutilization of NAC before RC to inform real‐world clinical practice. The present study has several limitations inherent to its design and the use of the NCDB. While it includes data for individuals diagnosed with urothelial carcinoma, the NCDB lacks detailed information on variant histological subtypes, which are established prognostic factors influencing treatment outcomes [48, 49, 50]. Thus, we were unable to study treatment patterns or outcomes within these subtypes. Additionally, the potential for misclassification of variant histologies further restricts the use of the NCDB to study these groups. Future research using datasets with more granular histological subtype data is necessary to address this gap. The NCDB exclusively captures data on de novo MIBC cases and does not include information on progressive or recurrent diseases, such as BCG‐unresponsive MIBC [51, 52, 53, 54]. As a result, treatment patterns and outcomes for these important subgroups could not be assessed. Longitudinal datasets with detailed treatment information, such as electronic health records (EHRs), are essential for evaluating these patient populations. The NCDB does not differentiate between robotic and open RC. A recent clinical trial indicates better 90‐day postoperative outcomes for robotic RC, potentially masking trends in postoperative outcomes among NAC users [44]. This lack of distinction limits our ability to further describe the observed trends in postoperative outcomes. We were unable to examine certain outcomes (e.g., survival information was not available for those diagnosed in 2017) given the limitations of the database. Longer‐term cancer outcomes, not typically studied in RCTs, will need to be investigated to generate more evidence about post‐treatment outcomes. These results are specific to the 2004 through 2017 time period, and 2017 was the most recent year of NCDB data available at the time that the study was initiated. Consequently, the results may not reflect the most recent trends in bladder cancer treatment, as clinical practices, treatment indications, and guidelines may have changed over this timeframe. Furthermore, the exclusion of more recent data, particularly from the post‐2017 period, presents a limitation given the rapidly evolving treatment landscape for MIBC. Despite these limitations, the NCDB is one of the largest hospital‐based cancer registries in the United States [19], and thus represents a unique and valuable source of generalizable information about treatment patterns and outcomes. Longitudinal datasets with more recent data and granular subtype information will be needed for a more complete investigation of treatment trends and outcomes in both de novo and progressive MIBC cases.

## 5 Conclusion

There has been a shift in treatment modalities for urothelial MIBC, with increased utilization of RC, trimodal bladder‐sparing approaches, and TURBT plus chemotherapy. The unmet need for NAC utilization and long‐term survival requires further investigation.

## Author Contributions

**Bernard Bright Davies‐Teye:** conceptualization (supporting), formal analysis (lead), investigation (equal), methodology (lead), project administration (equal), visualization (lead), writing – original draft (lead), writing – review and editing (lead). **M. Minhaj Siddiqui:** conceptualization (equal), supervision (equal), writing – review and editing (equal). **Xiao Zhang:** conceptualization (equal), supervision (equal), writing – review and editing (equal). **Abree Johnson:** conceptualization (equal), data curation (lead), formal analysis (supporting), investigation (equal), methodology (equal), project administration (equal), writing – review and editing (supporting). **Mehmet Burcu:** conceptualization (equal), funding acquisition (lead), investigation (equal), methodology (equal), writing – review and editing (equal). **Eberechukwu Onukwugha:** conceptualization (lead), data curation (lead), formal analysis (equal), investigation (equal), methodology (equal), project administration (equal), supervision (lead), validation (equal), visualization (equal), writing – review and editing (equal). **Nader Hanna:** conceptualization (equal), methodology (equal), supervision (equal), writing – review and editing (equal).

## Ethics Statement

This study was approved as nonhuman subjects research by the University of Maryland, Baltimore Institutional Review Board (HP‐00099619).

## Conflicts of Interest

Mehmet Burcu, BA, MS, PhD, FISPE, and Xiao Zhang, MD, DrPH are salaried employees of Merck Sharp and Dohme LLC, a subsidiary of Merck and Co. Inc., Rahway, NJ, United States.

## Disclaimer

The data used in the study are derived from a de‐identified NCDB file. The NCDB is a joint project of the Commission on Cancer (CoC) of the American College of Surgeons and the American Cancer Society. The CoC's NCDB and the hospitals participating in the CoC's NCDB are the sources of the de‐identified data used herein; they have not verified and are not responsible for the statistical validity of the data analysis or the conclusions derived by the authors.

## Data Availability

The data that support the findings of this study are available from the American College of Surgeons (ACS), but restrictions apply to the availability of these data, which were used under license for the current study, and so are not publicly available. Data are however available from the authors upon reasonable request and with permission of the [ACS].
